# Deciphering the Biological Enigma—Genomic Evolution Underlying Anhydrobiosis in the Phylum Tardigrada and the Chironomid *Polypedilum vanderplanki*

**DOI:** 10.3390/insects13060557

**Published:** 2022-06-19

**Authors:** Yuki Yoshida, Sae Tanaka

**Affiliations:** 1 Graduate School of Arts and Sciences, The University of Tokyo, 3-8-1 Komaba, Meguro-ku, Tokyo 153-8902, Japan; 2 Exploratory Research Center on Life and Living Systems (ExCELLS), National Institutes of Natural Sciences, 5-1 Higashiyama, Myodaiji, Okazaki 444-8787, Japan; 3 Institute for Advanced Biosciences, Keio University, 341-1 Mizukami, Tsuruoka 997-0052, Japan

**Keywords:** anhydrobiosis, tardigrades, chironomids, genomic evolution

## Abstract

**Simple Summary:**

Water is a requirement for life on Earth; loss of free water within the body or cell almost always leads to death. However, in several invertebrate lineages, some species can tolerate desiccation by entering an ametabolic state known as anhydrobiosis. Here, we review recent advances in our understanding of the molecular mechanisms and genomic evolution underpinning anhydrobiosis. We then propose several perspectives for further improving our understanding of anhydrobiosis.

**Abstract:**

Anhydrobiosis, an ametabolic dehydrated state triggered by water loss, is observed in several invertebrate lineages. Anhydrobiotes revive when rehydrated, and seem not to suffer the ultimately lethal cell damage that results from severe loss of water in other organisms. Here, we review the biochemical and genomic evidence that has revealed the protectant molecules, repair systems, and maintenance pathways associated with anhydrobiosis. We then introduce two lineages in which anhydrobiosis has evolved independently: Tardigrada, where anhydrobiosis characterizes many species within the phylum, and the genus *Polypedilum*, where anhydrobiosis occurs in only two species. Finally, we discuss the complexity of the evolution of anhydrobiosis within invertebrates based on current knowledge, and propose perspectives to enhance the understanding of anhydrobiosis.

## 1. Anhydrobiosis—A State with No Visible Signs of Life

Life on Earth depends on aqueous biochemical reactions [1], and approximately 70% of an animal’s body is composed of water [2]. Loss of water can cause incremental damage to biological systems at the cellular and tissue level. Therefore, most organisms take precautions against water loss by maintaining extra water or preventing water evaporation, depending on circumstances. However, several invertebrates have evolved a peculiar method of tolerating water loss. They can enter an almost completely dehydrated state when facing environmental desiccation and yet revive when rehydrated. This phenomenon is referred to as anhydrobiosis, a latent state of life induced by desiccation, first observed by Leeuwenhoek in 1702 [3]. Anhydrobiosis is considered to be a form of “cryptobiosis”, which is defined as “*the state of an organism when it shows no visible signs of life and when its metabolic activity becomes hardly measurable or comes reversibly to a standstill*” [4]. Metabolism is considered integral to life on earth, and hence cryptobiosis has been called a “third state of life” [5], alongside “life” and “death”. Today, anhydrobiosis has been observed in multiple invertebrate lineages, e.g., brine shrimps, bdelloid rotifers, nematodes, midges and tardigrades, as well as in the other kingdoms of Plantae, Fungi and Prokaryota (Table 1) [3,4,5,6,7,8,9,10]. Anhydrobiosis capabilities during life cycles vary between species; there are species that can undergo desiccation throughout their whole life cycle, whereas others can do so in a particular stage (i.e., eggs, larvae; Table 2) [11].

In addition to tolerating desiccation, organisms in the anhydrobiotic state can withstand exposure to other extreme chemical and physical conditions, a phenomenon known as cross-tolerance [30,31]. For example, tardigrades can tolerate low to high temperatures (−273–100 °C) [23,32,33,34,35], high pressure (7.5 GPa) and the vacuum of space [36,37,38,39], organic solvents [40], copper ions [41], salinity [42,43], hydrogen peroxide [44], and exposure to ultraviolet [45,46,47] and gamma [23,47,48,49,50,51,52,53,54,55,56] radiation. Interestingly, active tardigrade specimens can tolerate a high dose of gamma irradiation comparable to levels they withstand in the anhydrobiotic state (over 5000 Gy), suggesting the existence of a highly efficient DNA protection and repair system. Similar observations have been made in other anhydrobiotes, suggesting this cross-tolerance may be a common feature of anhydrobiotic organisms.

When organisms face severe water loss, damage occurs at various levels, affecting organ systems, tissues, cells and biomolecules [57,58,59]. For example, the exponential increase in ion concentration causes intense osmotic stress and oxidative stress, which can cause irreversible disruption of the lipid bilayer and protein structure, both of which rely on hydrophobic/hydrophilic interactions. This results in membranes becoming leaky and proteins partially unfolding, leading to aggregation. In addition, the stresses imposed by water loss may inhibit the activity of maintenance and repair pathways, negatively impacting the ability of the cell to remedy any damage to vital systems. Therefore, to achieve anhydrobiosis, a method of protecting and/or repairing biomolecules is required that also involves halting metabolism in the dry state and an efficient restart of cellular activity after rehydration.

Here, we review such molecular mechanisms of anhydrobiosis, from protective molecules to universal stress resistance pathways, to the underlying genomic evolution that has occurred in anhydrobiotes. We then introduce the evidence that has accumulated in the chironomid *Polypedilum vanderplanki* (Hinton, 1951) and phylum Tardigrada, where there are differences in anhydrobiotic machinery and protective molecules acquired. *Pol. vanderplanki* and its relative *Polypedilum pembai* (Cornette, 2017) are the only insects currently known to be capable of anhydrobiosis, which they achieve using canonical protective mechanisms, whereas anhydrobiosis has been observed throughout the phylum Tardigrada, where lineage-specific protein families seem to be important. Based on current knowledge, we discuss the peculiar enigma of anhydrobiosis and its molecular mechanisms.

## 2. Candidate Protective Molecules in Anhydrobiosis, from Trehalose to Intrinsically Disordered Proteins

### 2.1. “Traditional” Protective Molecules Accumulating in Anhydrobiotes

The current framework of cellular protection during anhydrobiosis in animals focuses on two elements: trehalose and late embryogenesis abundant (LEA) proteins. Both have been discovered as molecules whose accumulation is induced by desiccation, and are believed to contribute to anhydrobiosis.

Trehalose, a non-reducing disaccharide composed of two glucose molecules, is one of the most well-known compounds that contributes to the stabilization of biomolecules during desiccation and freezing, somewhat controversial in cells [60,61,62,63]. Several anhydrobiotic organisms such as yeast, nematodes, and *Pol. vanderplanki* accumulate trehalose to about 20% of their dry mass and rapidly degrade it upon rehydration (see Table 1) [12,20,64]. Since the 1970s, trehalose has been correlated with the survival of desiccation. Two hypotheses have been proposed for the molecular function of trehalose in cellular protection: vitrification and water replacement [21,65]. The vitrification model suggests that the glassy state formed by high concentrations of trehalose can prevent deleterious interaction between biomolecules. The water replacement hypothesis proposes that the O-H groups in trehalose form hydrogen bonds with hydrophilic groups (e.g., the phosphate head groups in phospholipids), in the same manner as water forms hydrogen bonds with biomolecules in bulk water, similarly preventing destructive interactions. These two hypotheses are not mutually exclusive, but whether either is, or both are, necessary for anhydrobiosis remains controversial.

A requirement for intra- and extracellular trehalose has been demonstrated in the budding yeast *Saccharomyces cerevisiae*, which accumulates approximately 400 mM trehalose inside the cell during the stationary phase and achieves 100% survival after lyophilization in a medium containing 500 mM trehalose [12]. Genetic studies show that the reduction in intracellular trehalose due to the disruption of the trehalose-6-phosphate synthase (TPS) gene attenuates tolerance of desiccation in the dauer larvae of the nematode *Caenorhabditis elegans*, and also in *S. cerevisiae* over long periods in the dry state, although trehalose is less important for short-term desiccation [13,14,15,17]. Additionally, approximately 16% of the Pv11 cell line (established from *Pol. vanderplanki* embryos) was found to survive desiccation after pre-incubation in a medium containing 600 mM trehalose [22,66]. This situation mimics the high concentration of trehalose dispersed throughout the whole body by the trehalose transporter 1 (TRET1) in *Pol. vanderplanki* larvae during entry into anhydrobiosis [67]. Apart from the possible protective functions discussed above, trehalose is also suggested to function as an energy source for successful recovery from anhydrobiosis [68].

On the other hand, tardigrades and bdelloid rotifers show no or minimal accumulation of trehalose during desiccation [19,69,70]. It has been demonstrated in the tardigrade *Richtersius coronifer* (Richters, 1903) that trehalose levels increase to approximately 2% during desiccation [69]. In addition, a comprehensive survey of Tardigrada showed that heterotardigrade species accumulated less than 0.05% of their dry weight as trehalose and less than 0.15–1% in eutardigrades (e.g., *Macrobiotus tonollii* (Ramazzotti, 1956), 1.65 ng/µg protein; a six-fold increase compared to the active state) [70,71]. High trehalose levels (70 ng/µg protein, percentage of dry weight not reported) were reported in *Paramacrobiotus metropolitanus* (Sugiura, Matsumoto and Kunieda, 2022) [26,72], but this is still less than reported in *Pol. vanderplanki* (see Table 1). Moreover, no substantial accumulation of any sugars was observed in a metabolomics study of the tardigrade *Ramazzottius varieornatus* (Bertolani and Kinchin, 1993) [71]. Taken together with the fact that several tardigrades have lost the genes for trehalose synthesis [26,73], it has been hypothesized that tardigrades may use trehalose partially in combination with other molecules, if at all, to achieve anhydrobiosis [74,75]. Trehalose might also be used in some species as a ‘safe’ means of storing energy: most sugar molecules, such as glucose, have a reducing group that can react with other biomolecules, but conversion to trehalose, which is non-reducing, prevents this reactivity while securing a carbohydrate energy source for use during recovery from anhydrobiosis or other stress conditions.

LEA proteins [76] have been of interest since the early molecular studies on anhydrobiosis machinery. This protein family is classified into seven groups [77,78] and was first identified in plant seeds, comprising up to 4% of total protein [79]. The LEA proteins found in the animal kingdom are mainly from group 3, as first reported in the entomopathogenic nematode *Steinernema feltiae* (Stanuszek, 1974) [80]. Orthologs in anhydrobiotic animals, such as bdelloid rotifers, brine shrimps and *Pol. vanderplanki*, as well as other nematodes, were identified among the genes induced by desiccation [81,82,83,84,85,86]. LEA proteins in aqueous solution are highly hydrophilic and unstructured, and remain soluble even after heat treatment; however, they can form amphipathic alpha-helices under water-deficit conditions, which is mimicked by tetrafluoroethylene (TFE) treatment in vitro. The alpha-helix is assumed to interact with the hydrophilic surface of proteins or lipids to prevent structural damage upon desiccation, which is referred to as the “*molecular shield model*” [77,87]. Indeed, LEA4 protein from *Pol. vanderplanki* prevents protein from aggregation caused by desiccation [88]. Moreover, the exogenous expression of LEA proteins in non-anhydrobiotic cells can confer moderate tolerance to hyperosmotic conditions [85,87,89,90]. Not only cytosolic LEA proteins, but also those localized to the mitochondria, e.g., RvLEAM (*R. varieornatus*) and AfrLEA3m (*Artemia franciscana*; Kellogg, 1906), can improve hyperosmotic tolerance in human cells [86,89]. These findings emphasize the contribution of LEA proteins to osmotic stress tolerance.

### 2.2. Abundant Proteins in an Anhydrobiotic Tardigrade Are Lineage-Specific Intrinsically Disordered Proteins

Unlike in other anhydrobiotes, LEA proteins are not extremely highly expressed nor accumulated to high levels in tardigrades, and therefore cannot be considered a major factor in tardigrade anhydrobiosis. During the quest to identify substitutes for trehalose and LEA proteins, methods to identify proteins with features similar to LEA proteins identified two highly heat-soluble multi-copy tardigrade-specific protein families: cytoplasmic abundant heat-soluble (CAHS) and secretory abundant heat-soluble (SAHS) proteins. These were among the most abundant proteins in the heat-soluble fraction extracted from *R. varieornatus* [91]. These proteins were named based on the subcellular localization of GFP-fused proteins in human cells; thus, CAHS proteins were localized to the cytoplasm or nuclei, while SAHS proteins were secreted into the culture medium. Although these two protein families do not show sequence similarity with canonical LEA proteins, they both form alpha-helical structures under water-deficit conditions or upon TFE treatment, as reported for LEA proteins. Moreover, since 2021, multiple studies have reported the formation of higher-order structures by CAHS proteins [92,93,94,95,96]. These studies argue that purified recombinant CAHS proteins form fiber-like aggregates or gel-like lumps at high concentrations, as is assumed to occur with the onset of desiccation. This phenomenon was also observed in bacteria and human cells expressing CAHS protein. This resembles what Tunnacliffe et al. had speculated while discussing the functional role of LEA proteins in 2005: “*If LEA proteins are able to form* *α-helical coiled coils on drying, they may also form higher-order supramolecular assemblies similarly to the way keratins, neurofilament proteins, and lamins, for example, form intermediate filaments*” [83]. Boothby et al. demonstrated that several CAHS proteins confer desiccation tolerance on bacteria and yeast, and the RNAi-mediated knock-down of CAHS transcripts in *H. exemplaris* reduces viability on recovery from anhydrobiosis [97]. This hypothesis is promising and intriguing, and thus the requirement for higher-order structures to form, rather than just the expression of CAHS proteins, should be validated in further analysis.

The successful identification of CAHS and SAHS proteins prompted a search for hydrophilic proteins with different subcellular localizations, which revealed two examples: tardigrade-specific mitochondrial abundant heat-soluble (MAHS) protein and canonical LEAM [86]. Both are highly expressed in tardigrades and, when expressed in human cells, localize to the mitochondria and confer improved osmotic tolerance. The anti-osmotic mechanism of MAHS protein has yet to be clarified, but the existence of two different heat-soluble proteins localizing to the mitochondria may indicate some functional specialization. An example of such specialization would be the heat-resistant obscure (Hero) proteins identified in *Drosophila* and human cell lines [98]. These proteins stabilize other proteins under stress conditions; however, they seem to prefer certain proteins over others. We speculate that MAHS and LEAM proteins may also show such “preferences” when protecting mitochondria. Further studies on protective specialization should focus not only on the localization of each protein within the mitochondria, but also on the basic properties of the proteins and the molecules being protected.

Furthermore, based on the hypothesis that high tolerance to radiation in the active state may be enabled by a protein that protects genomic DNA, the proteomic analysis of nuclei from *R. varieornatus* identified the intrinsically disordered damage suppressor (Dsup) protein [99]. As hypothesized, the Dsup protein prevents DNA damage induced by X-ray irradiation and H_2_O_2_ in cultured cells. Surprisingly, the Dsup protein binds to free DNA and/or chromatin and suppresses DNA damage by H_2_O_2_ in vitro [100]. The binding of Dsup to chromatin should provide insights into the protection mechanism of Dsup, and whether Dsup interferes with transcription and replication. A mammalian-specific nuclear protein, protamine, has been reported to replace histone proteins in sperm and possibly protect genomic DNA from cellular stress by condensing chromatin [101]. The similarity between Dsup and protamine may point to universal mechanisms that protect the genome from damage.

The proteins introduced above, e.g., LEA and CAHS proteins, are intrinsically disordered proteins (IDPs). In general, IDPs can have novel functions that might not be available to structured proteins, particularly under stress conditions; for example, the formation of condensates with RNA and/or other proteins in response to intracellular stress promoted by liquid–liquid phase separation (LLPS), which is thought to play an important protective role in anhydrobiosis [102]. It has been hypothesized that IDPs do not ordinarily form secondary structures, but they may do so under certain unusual conditions (i.e., extremes of pH, temperature and ion concentration, or due to changes in subcellular localization, etc.), resulting in novel interactions with other biomolecules. For example, the LEA6 protein of the brine shrimp *A. franciscana* undergoes LLPS during desiccation and maintains a vesicle-like structure in the dry state [102]. The IDP anhydrin, identified in the anhydrobiotic nematode *Aphelenchus avenae* (Bastian, 1865), has been reported to have both chaperone and endonuclease functions [103]. In future studies on IDPs, the complexity of their functions and under what conditions they function should be investigated, considering the possibility that an IDP might be multifunctional across several phases of the dehydration–rehydration cycle.

## 3. Conserved Cellular Maintenance and Repair Pathways Contributing to Anhydrobiosis

The utilization of the unique systems that enable anhydrobiosis is fascinating and often attracts attention, but anhydrobiosis machinery also comprises other elements. It is likely that protection by these proteins is not perfect; thus, damage to DNA and protein has been observed in specimens after rehydration, possibly due to reactive oxygen species (ROS) produced during the anhydrobiosis cycle [104,105,106]. Therefore, maintenance and repair mechanisms common to almost all living organisms are probably an essential component of anhydrobiosis machinery and help to restore cellular functions after rehydration.

The importance of antioxidant pathways has been emphasized in molecular [106,107,108] and omics analysis on anhydrobiosis [73,99,109,110,111,112,113,114,115,116,117]. The expression of superoxide dismutase (SOD), glutathione-S transferase (GST), and catalase is induced during desiccation in the tardigrade *Hypsibius exemplaris* (Gąsiorek, Stec, Morek and Michalczyk, 2018). Similarly, SOD, catalase, thioredoxin, and peroxidase are upregulated during desiccation in *Pol. vanderplanki* [113,118]. The importance of enhanced antioxidant capability in anhydrobiosis is emphasized by the expansion of relevant gene families in these genomes [73,99,109]. Interestingly, a novel manganese-dependent peroxidase family conserved widely throughout Tardigrada, as well as a catalase suggested to be of bacterial origin, have been identified in tardigrades [99,110]. However, it is unclear how and at what stage of the anhydrobiosis cycle ROS are generated (e.g., early and late entry, anhydrobiosis, early and late recovery), although it seems likely that the balance between the generation and detoxification of ROS is disrupted during desiccation due to perturbations in the cellular environment and the loss of water affecting the functionality of antioxidants.

ROS that evade antioxidant systems will damage cellular biomolecules, particularly DNA and proteins. DNA damage has been observed in chironomids, rotifers and tardigrades, and the damage incurred during anhydrobiosis was found to be of a similar level as that found in active chironomid larvae exposed to 70 Gy of heavy ion radiation [118,119,120,121]. Gene expression analysis has shown that many DNA repair pathways are induced in anhydrobiotes [73,116,118]. While we speculate that the Dsup protein can protect genomic DNA in *R. varieornatus*, DNA repair is clearly crucial in this species, as the MRE11 gene, whose protein participates in double-strand break repair, has been duplicated during evolution. These data underline the importance of DNA repair in anhydrobiosis. Furthermore, heat shock proteins (HSPs) are major contributors to the repair of protein damage. HSPs are known to be induced not only by heat treatment, but also by various other cellular stresses, e.g., UV radiation, osmotic stress and oxidative stress. They act as molecular chaperones to help with protein assembly and refolding. In tardigrades, it has been reported that HSP70 expression is higher in the rehydrated active state than before desiccation [122,123]. In contrast, in *Pol. vanderplanki*, the expression of several HSPs (e.g., HSP90s and HSP70s) is induced by desiccation [124]. Since HSPs are fundamental to the maintenance of cellular function, it is difficult to determine whether these proteins are a core element of anhydrobiosis machinery or part of a pathway that is activated as a result of damage caused by desiccation.

Thus, an important question concerning these proteins is whether they are regulated as part of anhydrobiosis machinery or whether they are induced by the detection of stress or damage in cellular molecules as a part of the normal cellular response. One way to answer this question is to identify how these pathways are regulated. Several signaling and transcription factors have been implicated in the regulation of anhydrobiosis machinery so far, including heat shock factor (HSF) and nuclear transcription factor Y subunit gamma-like (NF-YC) in *Pol. vanderplanki* [125,126], and AMP-activated protein kinase (AMPK), protein phosphatase 1 (PP1), and protein phosphatase 2A (PP2A) in *H. exemplaris* [24,127]. Trehalose secretion from fat body tissue is also seemingly a key signal of systemic anhydrobiosis in *Pol. vanderplanki* [66,67]. Although the molecular components of these two systems seem to differ, a comparison among anhydrobiotes might answer how these components are prepared, located and assembled, and how anhydrobiotic machinery is established.

## 4. Examples of Genomic Evolution Underlying Anhydrobiosis at Species (*Pol. vanderplanki*) and Phylum Levels (Tardigrada)

**Figure 1:**
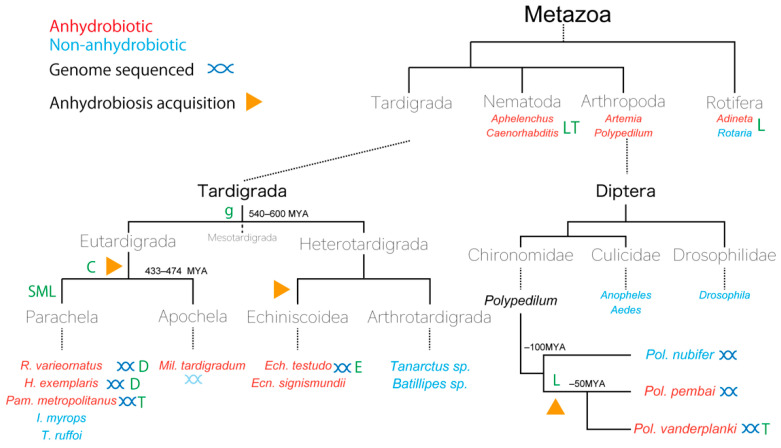
Distribution of anhydrobiosis and associated molecular mechanisms in invertebrates. Illustration of the phylogeny of anhydrobiotic species and major molecular mechanisms associated with them. The predicted emergence point of lineage-specific molecular species is indicated at the last common ancestor (i.e., CAHS proteins emerged somewhere before the divergence of Apochela and Parachela). Only lineages that contain anhydrobiotic species are indicated. Brown arrows indicate possible acquirement points of anhydrobiosis machinery. Green letter annotations indicate the conservation/utility of corresponding proteins/compounds: C: CAHS; S: SAHS; M: MAHS; L: LEA (includes LEAM); D: Dsup; g: Rv.g12777; T: Trehalose. Red and blue taxonomic labels indicate anhydrobiotic and non-anhydrobiotic capability, respectively.

*Pol. vanderplanki* and tardigrades are fascinating subjects for the study of anhydrobiosis; for both, a great deal of molecular data have accumulated in recent decades, as described in the previous sections. Two *Polypedilum* species (*Pol. vanderplanki* and *Pol. pembai*) are the only insects known to be capable of anhydrobiosis, and studies on *Pol. vanderplanki* have revealed the utilization of canonical mechanisms (e.g., trehalose and LEA proteins) to achieve anhydrobiosis, whereas tardigrades exploit novel proteins instead. Figure 1 shows the complexity of the evolution of anhydrobiotic capability, as well as the conservation of molecular mechanisms in tardigrades. In this section, we focus on these two invertebrate lineages to consider how anhydrobiotic capabilities have evolved.

### 4.1. Tardigrada: A Phylum Showing Complicated Evolution of Anhydrobiosis

The phylum Tardigrada comprises approximately 1,400 tardigrade species which are found in various environments including terrestrial, freshwater, and marine habitats [128,129]. These species are classified into two classes (see Figure 1): Eutardigrada, of which most species of both Apochela and Parachela orders are anhydrobiotic, and Heterotardigrada, which are further separated into the non-anhydrobiotic marine Arthotardigrada and mostly anhydrobiotic Echiniscoidea orders. A third class, Mesotardigrada, has been proposed, but it is considered *nomen dubium* following recent sampling efforts [130,131]. The common tardigrade ancestor is hypothesized to be of marine origin [132], suggesting that anhydrobiosis has been acquired twice, i.e., once in each lineage (Eutardigrada and Heterotardigrada), possibly due to selective pressure resulting from environmental fluctuation during terrestrialization [133]. Eutardigrada and Heterotardigrada are hypothesized to have diverged around 540–600 MYA [134,135], and Apochela and Parachela within Eutardigrada around 433 and 474 MYA [134], suggesting that the terrestrialization and acquisition of anhydrobiosis in Eutardigrada occurred during this 68–167-million-year period. Most molecular studies have focused on Eutardigrada species (e.g., *Milnesium tardigradum* (Doyère, 1840), *R. varieornatus*, and *H. exemplaris,* formerly known as *Hypsibius dujardini* (Doyère, 1840) [136]), for which rearing systems have been established [23,137,138]. Initial omics studies in tardigrades were conducted in *Mil. tardigradum*, a terrestrial anhydrobiotic Apochela species [139,140,141,142,143,144,145], and led to the first mechanistic model of anhydrobiosis in tardigrades [144].

It is known that anhydrobiotic capabilities vary between tardigrades species (Table 1). Thus, while marine heterotardigrades are thought to be incapable of anhydrobiosis (i.e., *Tanarctus* (Renaud-Debyser, 1959), *Batillipes* (Richters, 1909)), some non-marine species can survive immediate desiccation (i.e., *R. varieornatus* [23,146], *Mil. tardigradum* [27,147], and *Echiniscus testudo* (Doyère, 1840) [147]), while others must be desiccated gradually (i.e., *H. exemplaris* [24,136], *Pam*. *metropolitanus*). For example, *R. varieornatus* can directly enter anhydrobiosis at low relative humidity (37% RH) [23,148], whereas *H. exemplaris* requires 48 h preconditioning [24]. The inhibition of transcription and translation during preconditioning prevents a successful transition to anhydrobiosis [24], suggesting that de novo gene expression and/or production of protectants are essential in the latter species. Such requirements may be caused by differences in their habitats: *R. varieornatus* was isolated from moss on a bridge susceptible to repeated desiccation, whereas *H. exemplaris* was isolated from a lake. Additionally, several eutardigrade species (i.e., *Isohypsibius myrops* (Du Bois-Reymond Marcus, 1944) [149], *Thulinius ruffoi* (Bertolani, 1982) [150,151]) have lost their ability to undergo anhydrobiosis, which implies these species lack anhydrobiosis genes or the pathways that regulate these genes.

To decipher the complex evolution of tardigrade anhydrobiosis, a comprehensive comparative analysis based on genome information has been conducted between the two closely related tardigrades mentioned above, *R. varieornatus* and *H. exemplaris*, which show distinct anhydrobiotic entry modes [73,99]. The genomes of these species differ in size: *R. varieornatus* has a compact genome of 55.83 Mb, while that of *H. exemplaris* is 104.16 Mb [99]. A quantitative analysis of genome content indicates that approximately 80% of this nearly two-fold difference in size (38 Mbp out of 48.33 Mbp) is caused by an increase in the total length of repetitive elements (17 Mb) and in introns (21 Mb) in *H. exemplaris*, and not by whole-genome duplication. This difference was also observed between anhydrobiotic and non-anhydrobiotic bdelloid rotifers [152,153]. The gene content of *H. exemplaris* and *R. varieornatus* showed many similar characteristics in terms of the extensive duplication of antioxidative stress genes (e.g., SOD), loss of the peroxisomal oxidative pathway, stress response pathways (e.g., *Hif1a*, mTORC1), several genes within the DNA repair and telomere maintenance pathways, and the conservation of tardigrade-specific anhydrobiosis genes (i.e., CAHS, SAHS, MAHS, LEAM, and Dsup) [73,99,154,155]. On the other hand, gene expression analysis has indicated a difference between the two species; many tardigrade-specific anhydrobiosis genes are constitutively highly expressed in *R. varieornatus*, but are highly induced from basal levels in *H. exemplaris* [73,99]. These data suggest that the different modes of anhydrobiosis in these species are not the result of different genome content, but the differential expression of anhydrobiosis genes. Determining the pathways that regulate the expression of unique proteins and canonical maintenance pathways will provide insights into the genomic evolution of anhydrobiosis; in particular, a comparison of species that constitutively express anhydrobiotic genes and those that have secondarily lost the ability to enter anhydrobiosis may be of interest.

To expand our knowledge of genomic evolution within the phylum Tardigrada, the genome of a species within the class Heterotardigrada was recently reported [109]. The genome of *Ech. testudo*, a heterotardigrade that is capable of immediate transition into anhydrobiosis, revealed a more complicated picture of anhydrobiosis mechanisms in tardigrades, as none of the tardigrade-specific proteins identified in *R. varieornatus* were conserved. Given the fact that anhydrobiosis was independently acquired in eutardigrades and heterotardigrades, this might be understandable. The screening of highly expressed heat-soluble proteins identified the *Echiniscus testudo* abundant heat-soluble (EtAHS) protein family as possible analogs of the CAHS protein family [91]; however, there was no sequence homology between these two families. Similarly, the transcriptome of *Echiniscoides sigismundi* (Schultze, 1865) (Heterotardigrada) and the genome of Macrobiotoidea *Pam. metropolitanus* (Eutardigrada) showed the same conservation pattern (or lack of it) in tardigrade-specific anhydrobiosis genes, supporting the notion of the independent acquisition of protective proteins unique to tardigrades in these two lineages [26,112]. Further analysis of gene conservation in a wider range of lineages should help determine which elements of tardigrade anhydrobiosis are common and which are unique to particular lineages.

Recently, genomic analysis of the Apochela family, which solely consists of *Milnesium* species with strong anhydrobiotic capacity, has further added to the complexity of the evolution of anhydrobiosis in Tardigrada [156]. Although the genome of *Mil. tardigradum* was expected to have a gene content similar to Parachela species, only the CAHS proteins were conserved in *Milnesium*, indicating that other proteins, e.g., SAHS and MAHS proteins, may be limited to Parachela. The lack of SAHS and MAHS proteins may imply the existence of novel proteins or mechanisms that provide complementary functions in Apochela species able to undergo anhydrobiosis.

### 4.2. Polypedilum vanderplanki and Polypedilum pembai: The Only Insects Capable of Anhydrobiosis

**Figure 2:**
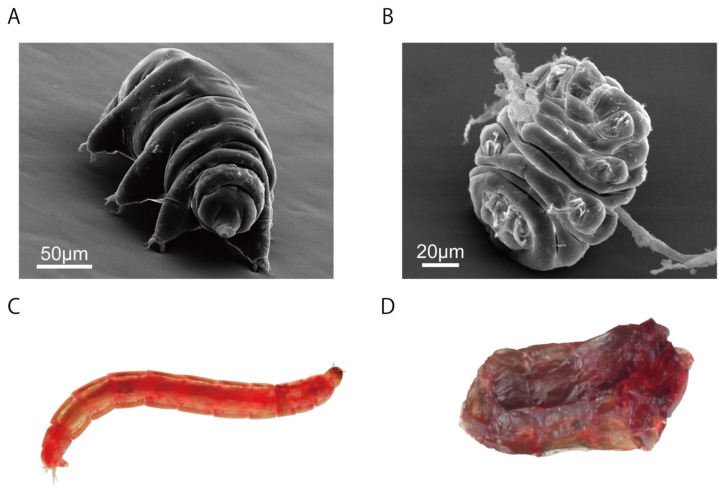
Tardigrades and chironomids. Images of *Macrobiotus shonaicus* (**A**,**B**) and *Polypedilum vanderplanki* (**C**,**D**) in the active state (**A**,**C**) and anhydrobiotic state (**B**,**D**). Images by Kenta Sugiura (**A**,**B**) and Gusev et al., 2014 (**C**,**D**) [114].

It has been estimated that there are nearly 10,000 chironomid species [157,158], making them one of the most diverse insect families. However, two chironomids, *Pol. vanderplanki* [8] and *Pol. pembai* [7], are the only species known within Insecta that can undergo anhydrobiosis (Figure 1 and Figure 2). Both midges can only enter anhydrobiosis at the larval stages [8], which represent 3–4 weeks (approximately 75%) of their life cycle [159]. These species inhabit semi-arid, rocky terrain in Africa [7,160], where they are subjected to desiccation in the dry season, such that the emergence of anhydrobiosis would represent a selective advantage. *Polypedilum nubifer* (Skuse, 1889), a closely related cosmopolitan species, is incapable of anhydrobiosis, and therefore anhydrobiosis must have been acquired during the ~50 MYA period when the *Pol. nubifer* and *Pol. pembai*–*Pol. vanderplanki* lineages diverged [7,114].

To identify genomic loci that are responsible for anhydrobiosis in *Pol. vanderplanki*, the draft genomes of *Pol. vanderplanki* and the cosmopolitan *Pol. nubifer* were reported and compared in 2014 [114]. Contrary to observations in tardigrades and bdelloid rotifers, these two species do not show large genome size differences; in fact, chironomids in general have a constant genome size of 100–200 Mbp [161]. Comparison between the *Pol. vanderplanki* and *Pol. nubifer* revealed duplications of multiple anhydrobiosis genes in the former species, i.e., those encoding LEA proteins, HSPs, GSTs, thioredoxins, etc. Interestingly, the authors identified nine genomic regions heavily enriched in these genes, which they dubbed Anhydrobiosis-Related Islands (ARIds). By expanding this genome analysis to the chromosome level, a subsequent study showed that seven of the nine ARIds were located on chromosome 4 in *Pol. vanderplanki*. Furthermore, a higher nucleotide diversity, the loss of synteny blocks with other chironomids, and the accumulation of novel gene families was also observed on chromosome 4, highlighting the importance of this chromosome in the evolution of anhydrobiosis in *Pol. vanderplanki* [162]. Additionally, a recent preprint described the genome of the anhydrobiotic *Pol. pembai*, which showed an independent evolution of ARId loci, harboring the multicopy PIMT gene family [163]. These data provide a fundamental hypothesis to explain the rapid genomic evolution of anhydrobiosis in *Pol. vanderplanki*.

Although only two *Polypedilum* species are capable of anhydrobiosis within the highly diverse Insecta, chironomids are found in various terrestrial and aquatic (both freshwater and marine) environments and have advanced into “niche” environments [164]. Therefore, there may be general genomic features that allow chironomids to adapt to fluctuating environments. A recent study reported a *Polypedilum* sp. as one of the dominant species in the Paraíba Basi (Brazil) during the extreme drought that occurred from 2012 to 2019 [165], possibly surviving through mechanisms similar to anhydrobiosis. The Brazilian chironomid may have a common ancestor with *Pol. vanderplanki* and *Pol. pembai,* and thus might represent a missing link between anhydrobiotic *Pol. vanderplanki* and non-anhydrobiotic *Pol. nubifer*. Information on the genome of this species will be instructive in this regard. Moreover, examining a wider taxonomic range would reveal whether there is a common factor, exemplified by chromosome 4 in *Pol. vanderplanki*, that underlies environmental adaptation in chironomids.

## 5. Considerations for Future Development in Anhydrobiosis Research

### 5.1. Complex Evolution Sometimes Calls for Elaborate Methods

The current research strategy for the dissection of the molecular mechanisms of anhydrobiosis depends heavily on comparative genomics to identify the key enabling gene repertoires. Therefore, the correct identification of functional orthologs of genes related to anhydrobiosis, alongside the basic analysis of genomic evolution, is required. For example, careful synteny analysis identified a homolog of the Dsup gene, initially identified in *R. varieornatus* and not in *H. exemplaris* by BLAST searches [73], which has extremely low similarity (bit-score 34.3, E-value = 0.09) to *Dsup* itself [154]. Although low conservation at the sequence level has also been observed in IDPs [166], no other tardigrade IDPs show the same degree of sequence diversity as Dsup. Such sequence diversity would hinder ortholog detection by sequence matching alone, and therefore synteny-based analysis has proven powerful in this context. In contrast, an example where synteny analysis did not work is *Pol. vanderplanki* chromosome 4. Although most of the *Pol. vanderplanki* genome shows synteny with the chromosomes of other mosquitoes, this was not the case for the genes on chromosome 4, suggesting extensive genetic turnover and recombination specific to this chromosome. Indeed, gene clustering analysis and annotation showed that numerous novel genes have accumulated on chromosome 4, causing the loss of synteny [162].

Another phenomenon that can confound comparative genomics is horizontal gene transfer (HGT). Novel gene acquisition by HGT is a rare event in eukaryotes [167] and is not more frequent in either tardigrades or chironomids than in other invertebrates [73,167]. However, genes encoding, for example, catalase, trehalose metabolic enzymes, UDP glycosyltransferases, and components of the ascorbate synthesis pathway are assumed to have arisen by HGT in tardigrades [26,73,99]. In chironomids, the multi-copy LEA protein gene family is hypothesized to result from HGT, with multiple duplications after genome integration [114], while in anhydrobiotic rotifers, HGT seems to have occurred on a much larger scale [152,168,169,170]. These findings suggest that HGT may have played an important role in the acquisition of anhydrobiosis, making the detection of horizontally transferred genes essential. However, homology-based HGT detection is prone to false positives, particularly in newly sequenced genomes, such that a thorough validation using additional methods is required [167].

In summary, sophisticated methods may be required in some cases, and may need to be fine-tuned for a more rigorous validation of hypotheses such as those indicated above. Thorough testing using phylogenetic analysis and additional confirmatory data (i.e., transcriptome assembly, protein-based analysis, etc.) is important [109]. Even with high-quality genomes, using basic homology-based searches for gene identification sometimes causes misinterpretations, as seen with the *H. exemplaris* genome [73,171,172,173,174,175,176].

To stimulate the production of new genomic datasets to enable high taxonomic coverage, the development of novel methods, alongside the existing technologies, will be required. A method enabling genome sequencing from a single individual has contributed to the removal of non-tardigrade contamination and should allow draft genomes for many tardigrade species to be obtained [110,173,177]. However, genome assemblies from short-read data alone will be highly fragmented, somewhat limiting genome analysis (for example, synteny analysis). Other methods that can be used are long-read sequencing and Hi-C; recent applications have produced pseudo-chromosomal genome assemblies in both chironomids (*Pol. vanderplanki* [162]) and tardigrades (*H. exemplaris* [178]). The application of these methods will likely improve taxonomic coverage, enabling a more thorough analysis of genomic evolution within Tardigrada.

### 5.2. Functional Analysis of Anhydrobiosis Genes In Vivo

As omics analyses yield new genes and proteins as candidates for roles in anhydrobiosis, functional analysis of these candidates, which might include protective molecules, maintenance pathways and regulatory pathways by loss- or gain-of-function experiments, will become increasingly necessary. In gain-of-function experiments, several genes have been expressed in desiccation-sensitive cells and species, such as human cells, yeast, plants and mice, to test for improved tolerance [86,91,97,99,110]. The tissue-specific expression of unique genes should be considered when the exogenous expression of gain-of-function analysis is performed. On the other hand, for loss-of-function experiments, RNAi-mediated gene knockdown has been established for *H. exemplaris* [179], and has demonstrated a decrease in the survival of anhydrobiosis after the inhibition of CAHS protein genes and antioxidant genes [97,106]. For *Pol. vanderplanki*, RNAi and CRISPR/Cas9 systems have been established in the anhydrobiotic cell line Pv11 [22,66,180]. However, in genomes with a significant expansion of key gene families, there is the problem of functional specification/redundancy and off-target effects as a result of the sequence similarity between gene copies, which might limit the efficacy of such approaches. Moreover, a problem unique to knockdown during anhydrobiosis is the need to consider the time required for RNAi to take effect; loss of water molecules during desiccation may affect transcription/translation capacity and RNAi efficiency.

### 5.3. Preconditioning Determines the Survival of Anhydrobiosis

Several tardigrade species, such as *R. varieornatus* and *Mil. tardigradum*, do not need to be prepared for entry into anhydrobiosis and they can undergo anhydrobiosis at any life stage, from embryos to adults [23,181]. On the other hand, other anhydrobiotic species, i.e., nematodes, chironomids and other tardigrades, need incubation for at least one day under high humidity conditions to sense impending desiccation and induce protection machinery [16,18,21,24]. Therefore, preconditioning methods are important when assessing anhydrobiotic ability, particularly when conducting functional assays of anhydrobiosis genes. However, anhydrobiosis induction methods vary between species and research groups (see Table 1). In *H. exemplaris*, all tardigrades incubated at 95% RH for four days survived subsequent desiccation [24], while only 2% survived after 16 h of preconditioning at 92% RH [25]. Similar differences in recovery from anhydrobiosis that depend on preconditioning conditions have also been observed in *Pol. vanderplanki* [21,182]. Such marked differences in survival rate emphasize the need for correct preconditioning treatments if anhydrobiosis is to be successful. It remains unknown whether “optimal” preconditioning can achieve near-zero cellular damage or whether such damage is unavoidable. Thus, there is a possibility that specimens that experience insufficient preconditioning, even with a 100% survival rate, may harbor cellular damage due to the “incomplete” assembly of anhydrobiosis machinery. To validate this, how different preconditioning treatments affect the expression of anhydrobiosis genes and the extent to which cellular damage occurs under these conditions should be correlated, while simultaneously taking into consideration the stages of anhydrobiosis (i.e., active, entry, anhydrobiosis, recovery, late recovery) and the difference in preconditioning requirements of each species (e.g., *R. varieornatus*, *H. exemplaris*). Such data would reveal whether repair systems are induced during rehydration from “successful” anhydrobiosis. Additionally, it should be noted that long-term preconditioning treatments may starve specimens, possibly inducing unexpected, potentially undesirable pathways as a consequence. These problems imply that the use of samples with inadequate preconditioning can lead to the misinterpretation of omics and biochemical studies. Although details of the mechanisms of anhydrobiosis are currently incomplete and seemingly complex, more extensive analysis may carve out a more simple model that defines the “essential core” of anhydrobiosis machinery.

### 5.4. Desiccation-Induced Quiescence and Cryptobiotic Anhydrobiosis

Anhydrobiosis is the ametabolic state induced by desiccation, while quiescence, a state which may seem similar, only reduces metabolic activity. The main difference is in the extent of metabolic activity; the desiccation-induced quiescent state shows signs of retaining some metabolic activity, which is absent in anhydrobiotic specimens. These two states have been mistaken in studies of anhydrobiosis. One study on *S. cerevisiae* reported the degradation of trehalose during long-term storage in a desiccation chamber [14]. However, this implies the yeast specimens were not in the anhydrobiotic state, because metabolism would not occur in cells with almost complete water loss. Another study reported that the *daf-2* mutant of *C. elegans* can survive exposure to 98% RH and 23% RH, but the survival rate at 0% RH decreased to 10%, even after pre-incubation at 98% RH for four days [17]. As anhydrobiotic specimens can survive at 0% RH for prolonged periods, these specimens may have been in a quiescent, rather than an anhydrobiotic, state. Unfortunately, there are no data to determine at what residual water content biological processes, such as metabolism, transcription and translation, halt as organisms or cells enter anhydrobiosis. Further studies that compare these two states, i.e., quiescence and anhydrobiosis, are required.

### 5.5. The Conundrum of Latent Life—Comparison of Distinct Anhydrobiotic Mechanisms across Phyla

Understanding the “true anhydrobiosis” [17] mechanism requires integrating evidence from anhydrobiotic species of different taxonomic groups, and this is what provoked our comparison of the accumulated knowledge on *Pol. vanderplanki* and tardigrades. In summary, we have listed the properties of anhydrobiosis in *Pol. vanderplanki* and three eutardigrade species (Table 2). The data from these taxa alone emphasize the complexity of anhydrobiosis, showing that tardigrades from the same class use markedly different machinery. Since anhydrobiosis occurs widely within phylum Tardigrada, it is anticipated that the overall picture of anhydrobiosis mechanisms in tardigrades will only become more complicated in the future.

As discussed above, anhydrobiosis raises intriguing issues in genomic evolution and biochemistry. Why do anhydrobiotic species appear sporadically in the animal kingdom? What advantage does a small genome size and the transition between the constitutive and de novo expression of anhydrobiotic genes have? Is trehalose essential for anhydrobiosis or can it be substituted? Why do several species enter anhydrobiosis only at a specific life stage, while others have this ability throughout their whole life cycle? Despite these important outstanding questions, we remain confident that the conundrum of latent life in true anhydrobiotic samples will be solved by the collection and analysis of more data, particularly given the advent of exciting new experimental approaches.

## Figures and Tables

**Table 1:** Survival of anhydrobiotes under various desiccation conditions.

Species	Desiccation Condition	Preconditioning	Survival Rate	Reference	Notes
Fungi					
*Saccharomyces* *cerevisiae*	Freeze-dryer for 3 days	2-week culture for stationary phase	~100% + 500 mM trehalose/~10% + 0 mM trehalose	Gadd et al., 1987 [12]	Intracellular trehalose was about 300 mM in the stationary phase
*Saccharomyces* *cerevisiae*	Air, 30 °C, ~16 h	72 h culture (late postdiauxic phase)	~50%, BY4741	Ratnakumar and Tunnacliffe, 2006 [13]	Intracellular trehalose was about 140 mM at the late postdiauxic phase
*Saccharomyces* *cerevisiae*	60% RH, 23 °C, > 48 h	5-day culture to saturation	<20%, WT, 2 days dry	Tapia and Koshland, 2014 [14]	Yeast had only 600 μg/mL trehalose after 5-day culture. Half of the trehalose degraded during the 30-day desiccation period, and more than 90% by 180 days
*Saccharomyces* *cerevisiae*	60% RH, 23 °C, >48 h	-	~1%, TDH3pr-AGT1, +1% trehalose	Tapia et al., 2015 [15]	AGT1 can transport extracellular trehalose. In 1% trehalose, intracellular trehalose was 157 μg/mL
Nematode					
*Aphelenchus avenae*	80% RH, 24 h; 40% RH, 24 h; 0% RH, 24 h	97% RH, 24–72 h	~50%	Higa et al., 1993 [16]	About 7% trehalose of dry weight under all preconditioning conditions
*Caenorhabditis* *elegans*	98% RH/23% RH/0% RH	98% RH, 4 days	~100%/~100%/~10%, *daf-2*	Erkut et al., 2011 [17]	Intracellular trehalose was about 400 mM after preconditioning
*Caenorhabditis* *elegans*	98% RH/23% RH/0% RH	-	~100%/~0%/~0%, *daf-2*	Erkut et al., 2011 [17]	Intracellular trehalose was about 80 mM without preconditioning
Rotifer					
*Adineta vaga*	22 °C, 7 days	In a container at 22 °C, 24 h	~80%, adults/~60%, juvenile/>80%, egg	Ricci, 1998 [18]	
*Philodina roseola*	Air (~33% RH), RT (~23 °C), 3 days	100% RH, 2 days	~75%, well fed	Lapinski and Tunnacliffe, 2003 [19]	Survival rate without preconditioning was less than 1%
Insect					
*Polypedilum vanderplanki*	<5% RH, RT (24–26 °C), >48 h	-	100%	Watanabe et al., 2002 [20]	Trehalose was 35 μg/individual at 48 h
*Polypedilum vanderplanki*	5% RH	100% RH for first day, 76% RH for the second day, and 5% RH for a third day	91%	Sakurai et al., 2008 [21]	The survival rate without preconditioning was 0%. Trehalose: 277 μg/mg dry weight with preconditioning; 4.2 μg/mg without preconditioning
*Polypedilum vanderplanki*, Pv11	<10% RH, 25 °C, +600 mM trehalose, >48 h	Incubation with 600 mM trehalose, 48 h	16%	Watanabe et al., 2016 [22]	
Tardigrade					
*Ramazzottius* *varieornatus*	0% RH, 25 °C, 10 days	85% RH, 25 °C, 24 h	~100%, egg, juvenile, and adult	Horikawa et al., 2008 [23]	
*Hypsibius* *exemplaris*	10%, RH 18 °C, 2 days	95% RH, 18 °C, 4 days	~100%	Kondo et al., 2015 [24]	For rehydration, specimens were transferred to 95% RH for 1 day
*Hypsibius* *exemplaris*	40% RH, 24 h; 22%, 7 days, 20 °C	92% RH, 20 °C, 16 h	~2%	Poprawa et al., 2022 [25]	
*Hypsibius* *exemplaris*	40–50% RH, 72 h; incubator,7 days, 20 °C	-	~50%	Poprawa et al., 2022 [25]	
*Paramacrobiotus* *metropolitanus*	10% RH, 22 °C, 2 days	95% RH, 22 °C, 48 h	>60%	Hara et al., 2022 [26]	Trehalose was 70 ng/μg protein after 2 days desiccation
*Milnesium* *tardigradum*	50–62% RH, 25 °C, 1 h	-	~90%	Horikawa and Higashi, 2004 [27]	
*Echiniscoides* *sigismundi*	62 or 39% RH, 22–23 °C, 48 h	-	~99%	Hygum et al., 2016 [28]	
*Richtersius* *coronifer*	65% RH, 23 °C, 12 days	-	~40%	Jönsson et al., 2001 [29]	

**Table 2:** Comparison between chironomid and tardigrades showing the current understanding of their anhydrobiotic mechanisms.

Species	Life Stage with Desiccation Tolerance	Trehalose Accumulation	IDP	Genome Size	Regulation of Anhydrobiotic Genes
*Polypedilum vanderplanki*	Only larva	35 μg/individual	LEA	104 Mb	Expression induction through HSF and NFY-C
*Ramazzottius varieornatus*	Embryo, juvenile, adult	300 μM/sample	CAHS, SAHS, MAHS, LEAM, Dsup	56 Mb	Constitutive expression
*Hypsibius* *exemplaris*	Adult	(gene lost)	CAHS, SAHS, MAHS, LEAM, Dsup	104 Mb	Regulation by AMPK and PP1/PP2A
(*Hypsibius* *dujardini*)					
*Paramacrobiotus* *metropolitanus*	Adult	70 ng/μg protein	CAHS, SAHS, MAHS, LEAM	170 Mb	-

## Data Availability

Not applicable.
